# Goal-directed bodily signals in birds and frogs

**DOI:** 10.3758/s13420-024-00640-5

**Published:** 2024-09-11

**Authors:** Kirsty E. Graham

**Affiliations:** 1 Department of Psychology, Hunter College, 695 Park Ave, New York, NY 10065 USA; 2 Wild Minds Lab, School of Psychology and Neuroscience, University of St Andrews, St Andrews, UK

**Keywords:** Communication, Intentionality, Gesture, Coordination, Sexual solicitation

## Abstract

Researchers have recently described the wing-fluttering signal of Japanese tits and eyeblink signal of concave-eared torrent frogs as bodily communication that elicits specific responses. I assess the evidence that these may be intentional, goal-directed signals using established criteria for gestural communication.

It is well established that primates, including humans, use gestures to communicate. Gestures are most often described as limb, head and body movements, and primates use them to achieve a range of social goals. But the study of gesture is not particularly inclusive of species with different body plans; although some researchers have adapted gestural methodologies to examine bodily communication in birds and fish (Ben Mocha et al., 2019; Vail et al., 2013). Before researchers leap to conclusions about the evolution of gesture and language, it is imperative to broaden research beyond primates. Here, I review two articles on bodily communication in nonhuman species – one on wing signals in Japanese tits (*Parus minor*; Suzuki & Sugita, 2024) and one on eyelid signals in concave-eared torrent frogs (*Odorrana tormota*; Chen et al., 2024). While they each take different approaches, both demonstrate bodily signals that might be considered goal-directed, in that they reliably precede a change in behaviour from the recipient.

In the first article, Suzuki and Sugita (2024) describe the “wing-fluttering” signal that Japanese tits perform outside of their nestbox that the researchers suggest is used to indicate to the bird’s partner to enter the nestbox first. This signal was used only when the signaller’s partner was within 5 m of the nest box (not when their partner was elsewhere); was used more often by females directed towards males; and was performed and almost always ended when the signaller’s partner entered the nestbox. When females did not flutter their wings, they usually entered the nestbox first, while when females fluttered their wings, their partner usually entered first. Males were quicker to enter the nestbox when their partner fluttered her wings than when she did not. Overall, the wing-fluttering action appears to change the behaviour of the recipient, and so may be considered goal-directed.

In the second article, Chen et al. (2024) describe the “eyeblink” behaviour that concave-eared torrent frog females perform before mating. The researchers conducted three studies: (1) wild observation; (2) two-choice amplexus experiment (amplexus is a copulatory embrace in which the male frog mounts and clasps the female); and (3) visual playback experiment. In the wild observation, three females exhibited frequent blinking before amplexus. In the two-choice amplexus experiment, females blinked significantly more before successful than before failed amplexus. And in the visual playback experiment, significantly more males preferred the blinking female video stimulus than the non-blinking stimulus. Blinking seems to be reliably associated with successful amplexus in this frog species.

One of the challenges of studying bodily communication is parsing communicative actions from physically similar functional actions. A chimpanzee can scratch their body to alleviate an itch, but they can also deploy a Big Loud Scratch/Exaggerated Scratch gesture to request that another individual groom them. Here we have two putative signals – wing-fluttering and eyeblinking – that resemble functional actions for these species. Birds can flutter their wings while moving or repositioning, and frogs (like many other animals) can blink to clean and lubricate their eyeballs. One way of determining whether these behaviours are communicative rather than functional would be to assess whether there are physical differences in how the signals are produced outside of these particular communicative contexts. For instance, is the angle and range of the fluttering movement different before entering the nestbox compared to a control period away from the nestbox? Is the duration of the blink different in a pre-mating context compared to non-mating contexts? These are also outstanding questions for primate gesture research (is the scratch louder, slower or more extensive when requesting grooming?), and so an open challenge to all studying bodily communication. Gathering baseline data on, for example, the morphology, duration and frequency of these behaviours in different contexts could also reveal other specific context(s) that share this signal use.

Both studies focused on the respective behaviour in a specific context and found evidence that the signal elicits a specific outcome. The wing-fluttering reliably predicts the partner entering the nestbox first, and eyeblinking reliably predicts successful amplexus. This could be a first indication that these are goal-directed signals. However, it will be necessary to follow up with studies assessing (a) how these signals meet other key criteria for intentionality, and (b) whether these behaviours could have alternative arousal-based explanations. Basically, these signals seem to provide information to the recipient, but an outstanding question is whether the signaller produces them with the goal to elicit a change in behaviour from the recipient.

If we apply the intentionality criteria outlined in Townsend et al. (2017) to each of these studies, we do find some evidence that these signals may be intentional. To be goal-directed, signallers should persist or elaborate with further signals, stopping when they have achieved the desired outcome. This was not reported in the torrent frogs, but Japanese tits stopped wing-fluttering once their partner entered the nestbox in 23 out of 26 cases. Both the birds and the frogs showed evidence of social use in terms of presence/absence of audience effect; for Japanese tits, no wing-fluttering was observed in the absence of a mate; for torrent frogs, females blinked more in the presence of a male during wild observations. Neither study addressed whether signals were affected by the composition of the audience or the behaviour of the audience. Sensitivity to the attentional state of the recipient was not reported for the frogs, but was found in all cases of wing-fluttering with the signallers’ “chests facing their mates from a location that should be visible to their mates” (Suzuki & Sugita, 2024). Manipulation of attentional state was not reported for frogs, and it is unclear from the description in the previous sentence whether the birds moved to face their chests to their mates, although it seems likely. Audience checking and gaze alternation was not reported in the birds, but might have occurred in the frogs with females “blink[ing] at the target male”, although there is no description of how researchers determined which was the target male (Chen et al., 2024). Finally, from the perspective of the recipient, for both birds and frogs the responses were “[r]epeatable, consistent and in line with the apparent intentions of the signaller” (Townsend et al., 2017).

While neither study directly addresses intentionality, both show some evidence of intentional communication according to criteria in Townsend et al. (2017). The missing criteria could likely be readily coded from the raw video data and may strengthen the case for considering that these signals are goal-directed. However, as has been argued for primate gesture research, it will also be important to rule out arousal-based explanations for these behaviours. If frogs blink more when sexually aroused, blinking might be a reliable indicator to male frogs that the female is sexually aroused and receptive – and so the blinking would have the effect of receiving a response without having been an intentional signal. If waiting outside a nestbox is stressful, it could be possible that fluttering is a stress response that delays the individual’s entry. Hormonal studies may help in assessing the likelihood of arousal-based explanations for these signals.

Nevertheless, both articles readily lay out their limitations and future directions, and so I look forward to follow-up research on these communication systems. The study of intentional bodily communication has largely focussed on primates and so each new study from other species vastly broadens our knowledge. Frameworks like the one offered by Townsend et al. (2017) are invaluable tools for comparative cognition research, allowing us to assess whether we are finding truly shared abilities across species. Whether Japanese tit wing-fluttering and concave-eared torrent frog eyeblinking are goal-directed, intentional signals remains partially unsolved (Chen et al., 2024; Suzuki & Sugita, 2024), but the evidence presented in these studies is positive and there are clear pathways for filling in the gaps.

## Data Availability

Not applicable.
